# Predicting Mortality among Hospitalized Children with Respiratory Illness in Western Kenya, 2009–2012

**DOI:** 10.1371/journal.pone.0092968

**Published:** 2014-03-25

**Authors:** Gideon O. Emukule, Meredith McMorrow, Chulie Ulloa, Sammy Khagayi, Henry N. Njuguna, Deron Burton, Joel M. Montgomery, Philip Muthoka, Mark A. Katz, Robert F. Breiman, Joshua A. Mott

**Affiliations:** 1 Kenya Medical Research Institute/Centers for Disease Control and Prevention-Kenya (KEMRI/CDC), Nairobi and Kisumu, Kenya; 2 Influenza Division, National Center for Immunization and Respiratory Diseases, U.S. Centers for Disease Control and Prevention, Atlanta, Georgia, United States of America; 3 Stanford University School of Medicine, Stanford, California, United States of America; 4 Ministry of Public Health and Sanitation, Division of Disease Surveillance and Response, Nairobi, Kenya; 5 Centers for Disease Control and Prevention, Port-au-Prince, Haiti; 6 Emory University, Atlanta, Georgia, United States of America; University of California, San Francisco, United States of America

## Abstract

**Background:**

Pediatric respiratory disease is a major cause of morbidity and mortality in the developing world. We evaluated a modified respiratory index of severity in children (mRISC) scoring system as a standard tool to identify children at greater risk of death from respiratory illness in Kenya.

**Materials and Methods:**

We analyzed data from children <5 years old who were hospitalized with respiratory illness at Siaya District Hospital from 2009–2012. We used a multivariable logistic regression model to identify patient characteristics predictive for in-hospital mortality. Model discrimination was evaluated using the concordance statistic. Using bootstrap samples, we re-estimated the coefficients and the optimism of the model. The mRISC score for each child was developed by adding up the points assigned to each factor associated with mortality based on the coefficients in the multivariable model.

**Results:**

We analyzed data from 3,581 children hospitalized with respiratory illness; including 218 (6%) who died. Low weight-for-age [adjusted odds ratio (aOR) = 2.1; 95% CI 1.3–3.2], very low weight-for-age (aOR = 3.8; 95% CI 2.7–5.4), caretaker-reported history of unconsciousness (aOR = 2.3; 95% CI 1.6–3.4), inability to drink or breastfeed (aOR = 1.8; 95% CI 1.2–2.8), chest wall in-drawing (aOR = 2.2; 95% CI 1.5–3.1), and being not fully conscious on physical exam (aOR = 8.0; 95% CI 5.1–12.6) were independently associated with mortality. The positive predictive value for mortality increased with increasing mRISC scores.

**Conclusions:**

A modified RISC scoring system based on a set of easily measurable clinical features at admission was able to identify children at greater risk of death from respiratory illness in Kenya.

## Introduction

Pediatric respiratory disease is a major cause of morbidity and mortality in the developing world with 70% of the global deaths occurring in Africa and Southeast Asia [1]–[4]. In Kenya, 16% of annual deaths in children <5 years of age are attributed pneumonia [5].

Lack of early and accurate detection of high-risk patients contributes to preventable complications and deaths associated with respiratory illnesses [6]. The limited diagnostic and treatment options available in most hospitals in developing countries have led to the development of syndrome-based guidelines for care. In 1999, the Kenyan Government introduced the Integrated Management of Childhood Illness (IMCI) to standardize and improve treatment for major causes of death in children <5 years [7]. Although implementation of IMCI remains suboptimal in Africa [8], IMCI includes guidelines to help health care workers identify “danger signs” for children at risk of severe illness who should be hospitalized [9].

Whereas IMCI seeks to identify young infants and children in need of hospitalization in developing country settings, no guidelines exist to help health care workers identify children who are at higher risk of dying from respiratory infections within the hospital setting. Recently, Reed and colleagues developed a Respiratory Index of Severity in Children (RISC) scoring system to quantify the severity of pediatric pneumonia for HIV positive and HIV negative children aged <24 months in South Africa [10]. This system has not been validated in other lower-resource countries in tropical sub-Saharan Africa. We used a similar methodology to create a modified Respiratory Index of Severity in Children (mRISC) scoring system for children under age five who were hospitalized with a respiratory illness in a government referral hospital in western Kenya.

## Methods

### Study Site and Population

We conducted our study among children <5 years old hospitalized with severe acute respiratory illness (SARI) at Siaya District Hospital (SDH) between August 2009 and July 2012. SDH is located in Nyanza Province in Western Kenya, and is an outpatient and inpatient facility with a bed capacity of 200. As a study site for the Kenya Medical Research Institute (KEMRI), other evaluations of public health interventions take place at SDH, including ongoing malaria and TB vaccine and treatment studies.

The population of Siaya district is almost entirely rural [11]. The area is holoendemic for malaria and has a high child mortality rate [11]. In 2007, 15% of the population in Nyanza aged between 15 and 64 years were infected with HIV [12].

### Data Collection and Case Definitions

Since August 2009, KEMRI and the United States Centers for Disease Control and Prevention (U.S. CDC) have been conducting hospital-based surveillance for SARI at SDH. Trained surveillance officers (mostly nurses) enrolled all consenting patients who were admitted with SARI at SDH. For minors, written informed consent was obtained from their parent or guardian. SARI was defined as an acute onset of cough or difficulty breathing within the last 14 days requiring hospitalization.

Demographic, clinical signs and symptoms, vital signs, comorbidity, and height/weight anthropometric data were recorded using a structured questionnaire using methods previously described [13]. The parent or caregiver accompanying the child provided information on history of present illness at the time of admission. They also provided information on recent hospitalizations for similar symptoms, cough, convulsions, lethargy, altered mental status, diarrhea, vomiting and difficulty feeding/breastfeeding in the last 14 days.

We used the following physical exam findings to assess respiratory distress: nasal flaring, stridor at rest, wheezing, chest wall in-drawing, and level of consciousness, indicated by being alert and awake, responding to voice commands, responding to mild pain and unresponsive/unconscious [AVPU (“alert, voice, pain, unresponsive”) scale]. We used the WHO IMCI algorithm to assess axillary temperature and age-based respiratory rate. Children were considered to have low oxygen saturation if a pulse oximetry reading (collected routinely) on room air was ≤90%. SARI patients were also classified as having no pneumonia, non-severe, severe or very severe pneumonia according to IMCI guidelines [14].

Based on WHO z-scores [15], three growth standards were evaluated: weight for age, weight for length, and length for age.

### Laboratory Methods

All patients admitted to the hospital for any reason were offered voluntary counseling and testing for HIV. HIV testing procedures at SDH have been described previously [16]. Children with both known and unknown HIV status were included in this analysis. All children <5 years who were admitted to SDH were also screened for malaria using screening methods previously described [17].

### Statistical Analyses

#### Identifying factors associated with mortality

We used bivariate and multivariable logistic regression models to assess relationships between demographic characteristics, co-morbidities, clinical signs and symptoms and in-hospital mortality. Odds ratios and 95% confidence intervals (CI) for mortality, as well as p-values, were calculated for each variable assessed. Variables with a p-value <0.2 in bivariate analysis were considered for the multivariable model. All two-way interactions were considered. We used the stepwise backward selection method to select the variables that were significant at p<0.05 in the multivariable model. The final selected model included only variables that were significant at p<0.05, and/or those variables that if removed would significantly increase the −2 log likelihood of the model [18], [19]. We considered participation in other ongoing clinical studies, treatment with anti-malarials, or treatment with anti-retrovirals (ARV) as potential confounders.

Age was re-coded into the following age groups for analysis: 0–2 months, 3–11 months, 12–23 months and 24–59 months. Measures of alertness reflecting “not fully alert” [only responsive to stimuli (voice or pain) or unresponsive] were grouped together and compared against those who were fully alert (i.e. fully alert on physical exam vs. not fully alert). Records with incomplete data (missing data on the variables assessed) were not included in the analysis.

#### Developing a scoring system

A point-based scoring system was developed from the final multivariable logistic regression model. Participation in clinical research studies and type of treatment provided during hospitalization were adjusted for, if indicated to be potential confounders, but not included in the development of the scoring system. Points were assigned to each variable associated with mortality in the model by rounding each β coefficient estimate to the nearest integer [10]. By adding up the points assigned to each predictive variable, a score was given to each patient, with a higher score corresponding to a greater risk for mortality. The mRISC scores ranged from a minimum of −2 to a maximum of 7 points. We calculated the sensitivity, specificity and positive predictive value (PPV) and negative predictive value (NPV) for mortality at different thresholds of mRISC scores. Scores <0 were re-coded as 0.

#### Statistical validation

We used a random sample of two thirds (67%) of the records in our dataset (“training dataset”) to develop the prediction model and compared the discrimination and calibration of predictions of the resulting model in the remaining one third (33%) of the records (“validation dataset”). The final model fitted using the training dataset performed equally well in the validation dataset as evidenced by the optimism-corrected C-statistic and goodness of fit test. The optimism-corrected C-statistic was 0.835 in the training dataset compared to 0.880 in the validation dataset and the goodness-of-fit tests had p = 0.650 and p = 0.336 in the training and validation datasets, respectively. Based on these findings, we used the entire dataset to develop the final model.

A number of diagnostic procedures were used to assess the fit and the predictive power of the final model. This model excluded the covariates measuring participation in ongoing clinical studies (previously adjusted for as potential confounders) as they could limit generalizability of the predictive power of the model to other settings. Model calibration (extent of bias) was assessed using the Hosmer and Lemeshow goodness-of-fit test [18]. We additionally also plotted predicted mortality probabilities and observed mortality against the mRISC scores. The predicted deaths were obtained by averaging the fitted values from the final selected model for each mRISC score. This graphical assessment was also performed on two subsets of the datasets divided into two equal time periods of 18 months (August, 2009 to January 2011 and February 2011 to July 2012) to evaluate if the model fitted data equally well for data collected at these different times. We also applied this graphical assessment to children <2 years and those aged two up to <5 years [10].

Discrimination was assessed using the area under the receiver operating characteristic (ROC) curve also known as the concordance (or *c*) statistic [20], [21]. Internal validity of the model was assessed using data from the same population. Using bootstrap samples, with 500 replications of the same size of the original dataset drawn with replacement, we estimated the optimism of the model [22]. The multivariable model was re-estimated in each of the bootstrap samples, each time calculating the difference between the c-statistic in the bootstrap sample and the one in the original data. The optimism was calculated as the average of these differences over the 500 bootstrap replications. The estimated model discrimination was then calculated by subtracting the average optimism from the c-statistic estimated from the original data and reported with the corresponding 95% CI [21]. All statistical analyses were performed using Stata version 12.1 (Stata Corp. 2011. *Stata Statistical Software: Release 12*. College Station, TX: Stata Corp LP).

### Ethical Considerations

This study was approved by the institutional review board of the U.S. CDC (CDC-3308) and the ethical review committee of KEMRI (SSC-1801). Written informed consent was obtained from all participants or caretakers/guardians of all minors prior to enrolment in the study.

## Results

**Figure 1:**
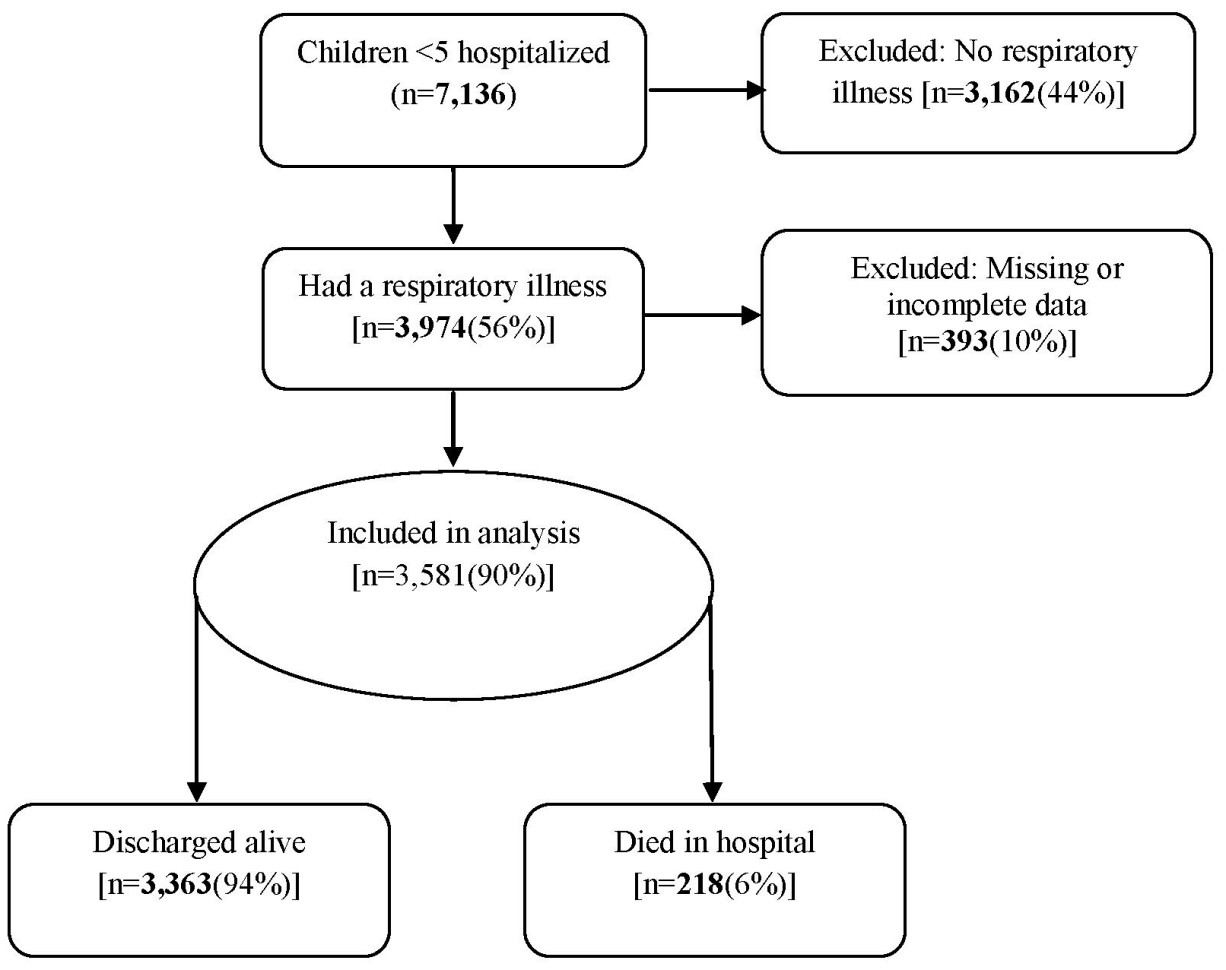
Children included in, and excluded from, the evaluation of mRISC score.

During August 2009 to July 2012, 3974 children aged <5 years were hospitalized at SDH with SARI, representing 56% of all hospitalizations among children under age 5 at SDH during this time period. Ninety percent (3581/3974) of children had complete data on the variables of interest and were included in the analysis (Figure 1). When the 393 excluded patients were compared with those who were included in the analysis, no significant differences were seen in terms of demographic characteristics or mortality. Death was the outcome for 218 (6%) patients. The median length of stay for all SARI admissions was 3 days [inter quartile range (IQR) = 2–5 days] but was significantly shorter for those who died [median = 1 day; p<0.001]. Most (75%) of the children aged <5 years and admitted with SARI were aged <2 years of age. Although HIV testing was offered to all admitted patients, 1322 (37%) were not tested. Of the 2259 children who had ever been tested for HIV, 198 (9%) were HIV-infected, and among them 130 (66%) had either been enrolled in HIV care (provided cotrimoxazole) or started on ARVs. A total of 2028 (56%) and 388 (11%) of the admissions had IMCI-defined pneumonia and severe/very severe pneumonia respectively. Of the children who died in the hospital, 67 (31%) had IMCI-defined severe/very severe pneumonia upon admission (Table 1).

**Table 1:** Bivariate associations between demographic characteristics, hospital admission clinical signs and clinical symptoms and mortality among children <5 years hospitalized with a respiratory illness in Siaya District Hospital, August 2009–July 2012.

Variable¥	Admissions(N = 3,581)	Deaths(N = 218)	OR(95% CI)	p-value
	n (%)	n (%)		
Age				
* 0–2 months*	136 (3.8)	13 (6.0)	2.1 (1.1–4.1)	0.023
* 3–12 months*	1,572 (43.9)	118 (54.1)	1.6 (1.1–2.3)	0.008
* 13–23 months*	987 (27.6)	45 (20.6)	1.0 (0.6–1.5)	0.852
* 24–59 months*	886 (24.7)	42 (19.3)	Ref	
Enrolled in clinical research studiesŦ	931 (26.0)	17 (7.8)	0.2 (0.1–0.3)	<0.001
Lab confirmed malaria	1,573 (43.9)	49 (22.5)	0.3 (0.3–0.5)	<0.001
Tuberculosis (On TB treatment ordiagnosed TB case)	102 (2.8)	17 (7.8)	3.3 (1.9–5.6)	<0.001
HIV status				
* Negative*	2,061 (57.6)	86 (39.4)	Ref	
* Positive*	198 (5.5)	25 (11.5)	3.3 (2.1–5.3)	<0.001
* Unknown*	1,322 (36.9)	107 (49.1)	2.0 (1.5–2.7)	<0.001
Weight for age				
* Low(−3<z-score*≤*−2)*	506 (14.1)	40 (18.3)	2.3 (1.5–3.3)	<0.001
* Very low(z-score≤−3)*	566 (15.8)	86 (39.4)	4.7 (3.5–6.4)	<0.001
Height/Length for age				
* Low(−3<z-score*≤*−2)*	548 (15.3)	32 (14.7)	1.2 (0.8–1.8)	0.338
* Very low(z-score≤−3)*	619 (17.3)	69 (31.7)	2.5 (1.8–3.4)	<0.001
Weight for Height/Length				
* Low(−3<z-score*≤*−2)*	388 (10.8)	28 (12.8)	1.7 (1.1–2.7)	0.010
* Very low(z-score≤−3)*	471 (13.2)	74 (33.9)	4.2 (3.1–5.7)	<0.001
Non-severe pneumonia^a^	2,028 (56.6)	77 (35.3)	0.4 (0.3–0.5)	<0.001
Severe pneumonia or very severe disease^b^	388 (10.8)	67 (30.7)	4.2 (3.1–5.7)	<0.001
Dehydration	449 (12.5)	60 (27.5)	2.9 (2.1–4.0)	<0.001
Previous hospitalization with the same illness	43 (1.2)	10 (4.6)	4.9 (2.4–10.0)	<0.001
Was unconscious	398 (11.1)	56 (25.7)	3.1 (2.2–4.2)	<0.001
Been lethargic	1,657 (46.3)	137 (62.8)	2.1 (1.5–2.7)	<0.001
Had diarrhea	1,619 (45.2)	128 (58.7)	1.8 (1.4–2.4)	<0.001
Unable to drink/breastfeed	338 (9.4)	50 (22.9)	3.2 (2.3–4.5)	<0.001
Night sweats	2,434 (68.0)	99 (45.4)	0.4 (0.3–0.5)	<0.001
Elevated respiratory rate for age^c^	2,278 (63.6)	118 (54.1)	0.7 (0.5–0.9)	0.003
O2 saturation (<90%)	574 (16.0)	54 (24.8)	1.8 (1.3–2.5)	<0.001
Chest wall in-drawing	940 (26.2)	119 (54.6)	3.7 (2.8–4.9)	<0.001
Nasal flaring	1,001 (28.0)	120 (55.0)	3.4 (2.6–4.6)	<0.001
Stridor at rest	213 (5.9)	29 (13.3)	2.7 (1.7–4.0)	<0.001
Wheezing on exam	143 (4.0)	18 (8.3)	2.3 (1.4–3.9)	0.001
A.V.P.U scale - Not alert^d^	128 (3.6)	52 (23.9)	13.5 (9.2–19.9)	<0.001
Treated with antimalarials	929 (26.0)	30 (13.8)	0.4 (0.3–0.6)	<0.001
On Septrin or ARVs	130 (3.6)	14 (6.4)	1.9 (1.1–3.4)	0.025

a Defined as per the WHO IMCI case definition: Cough or difficult breathing and elevated respiratory rate;

b Defined as per the WHO IMCI case definition: Cough or difficult breathing and any general danger sign or chest in-drawing or stridor in calm child;

c Elevated respiratory rate for age based on WHO IMCI algorithm; <2 months, >60 breaths/minute; 2–11 months, >50 breaths/minute;12–59 months, >40 breaths/minute;

d Combines responds to voice commands, responds to mild pain and unresponsive/unconscious;

*All admissions including deaths;

Ŧ Enrolled in research studies conducted by KEMRI/CDC (Malaria and TB studies);

¥ Variables that were not statistically significant at α = 0.05 are not shown on the table.

### Model Building and Score Development

Most of the variables assessed with the exception of sex, history of vomiting and convulsions, abnormal temperature (<35°C or ≥38°C), and an admission diagnosis of sepsis and/or bacteremia and anemia, were significantly associated with mortality in the bivariate analysis (Table 1).

Participation in ongoing clinical studies (aOR = 0.2; 95% CI 0.1–0.4) and treatment with anti-malarials (aOR = 0.5; 95% CI 0.3–0.7; p = 0.001) were both negatively associated with mortality and were adjusted for in the final multivariable model. Treatment with cotrimoxazole or ARVs was significantly associated with mortality in the bivariate analysis, but not in the multivariable model and was excluded from the final model.

In the multivariable model, seven independent variables were associated with mortality: low (−3<z-score≤−2) weight-for-age (aOR = 2.1; 95% CI 1.3–3.2), very low (z-score≤−3) weight-for-age (aOR = 3.8; 95% CI 2.7–5.4), an admission diagnosis of dehydration (aOR = 1.9; 95% CI 1.3–2.8), caretaker-reported history of unconsciousness during current illness (aOR = 2.3; 95% CI 1.6–3.4), inability to drink or breastfeed (aOR = 1.8; 95% CI 1.2–2.8), chest wall in-drawing (aOR = 2.2; 95% CI 1.5–3.1), and not being fully alert on physical exam (aOR = 8.0; 95% CI 5.1–12.6), (Table 2). Laboratory-confirmed malaria (aOR = 0.2; 95% CI 0.1–0.4) and history of night sweats during current illness (aOR = 0.5; 95% CI 0.3–0.7) were negatively associated with mortality (Table 2). Independent from the presence of malaria or chest wall in-drawing, having both malaria and chest wall in-drawing (interaction term) was also associated with increased risk of mortality (aOR = 3.6; 95% CI 1.7–7.8).

**Table 2:** Factors associated with mortality among children <5 years hospitalized with a respiratory illness in Siaya District Hospital, August 2009–July 2012.

Variable	AdjustedŦ OR (95% CI)	p-value	Coefficient Estimate (β)	Score
Lab confirmed Malaria	0.2 (0.1–0.4)	<0.001	−1.48	−1
Weight for age				
*Low(−3<z-score*≤*−2)*	2.1 (1.3–3.2)	0.001	0.73	1
*Very low(z-score≤−3)*	3.8 (2.7–5.4)	<0.001	1.34	1
Dehydration	1.9 (1.3–2.8)	0.001	0.64	1
Was unconscious	2.3 (1.6–3.4)	<0.001	0.83	1
Unable to drink/breastfeed	1.8 (1.2–2.8)	0.003	0.61	1
Night sweats	0.5 (0.3–0.7)	<0.001	−0.74	−1
Chest wall in-drawing	2.2 (1.5–3.1)	<0.001	0.78	1
Lab confirmed malaria* chest wall in-drawing^a^	3.6 (1.7–7.8)	0.001	1.29	1
A.V.P.U scale - Not alert^b^	8.0 (5.1–12.6)	<0.001	2.08	2

a Interaction between malaria and chest wall in-drawing;

b Combines responds to voice commands, responds to mild pain and unresponsive/unconscious;

Ŧ Adjusted for enrollment in clinical research studies, treatment.

A sub analysis of the 2695 (75%) children aged <2 years and 886 (25%) children aged ≥2 years hospitalized with a respiratory illness showed that chest wall in-drawing on physical exam, reported inability to drink or breastfeed at time of examination, and admission diagnosis of dehydration were only significantly associated with mortality among children <2 years but not in their older counterparts.

### Sensitivity and Specificity of the Scoring Model

Table 3 shows the sensitivity, specificity, PPV and NPV for predicting mortality at various cut-off points of the mRISC score. The PPV for mortality increased with increasing mRISC score from 6% at a cut-off score of 0 to 80% for patients with a cut-off score of 6. Of the 176 children who were admitted with an mRISC score of 3 or more, 70 (40%) died in the hospital. Similarly, 31/55 (56%) and 4/5 (80%) of those who were admitted with mRISC scores of ≥4 and ≥6 respectively, died in the hospital.

**Table 3:** Distribution of mRISC scores and screening performance in children <5 years hospitalized with respiratory illness in Siaya District Hospital, August 2009–July 2012.

mRISC Score	Patients	Actual deaths (%)	Sensitivity (%)	Specificity (%)	Positive Predictive Value (%)	Negative Predictive Value (%)
≥0Ŧ	3,581	218 (6.1)	100.0	0.0	6.1	
≥1	1,089	176 (16.2)	80.7	72.9	16.2	98.3
≥2	445	119 (26.7)	54.6	90.3	26.7	96.8
≥3	176	70 (39.8)	32.1	96.9	39.8	95.7
≥4	55	31 (56.4)	14.2	99.3	56.4	94.7
≥5	15	11 (73.3)	5.1	99.9	73.3	94.2
≥6	5	4 (80.0)	1.8	99.9	80.0	94.0

Ŧ Scores <0 recoded as 0.

Four percent (95% CI 3.0–4.6) of children admitted with IMCI non-severe pneumonia died in the hospital, with a PPV and NPV of 4% and 91% respectively for mortality. The PPV of IMCI-defined severe/very severe pneumonia for mortality was 17%.

### Statistical Validation

**Figure 2:**
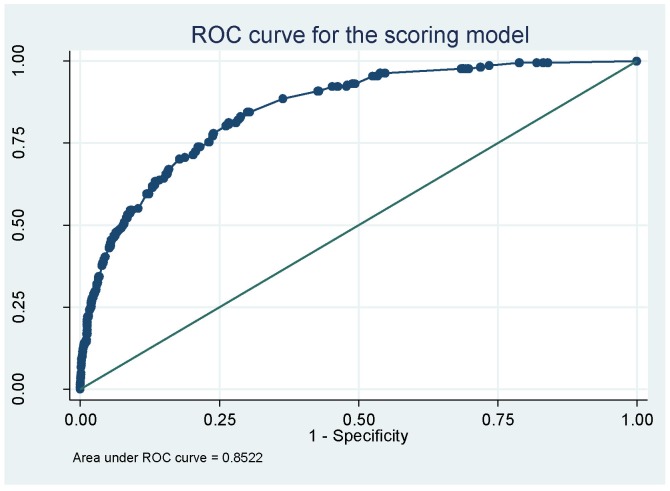
Receiver operating curve (ROC) for the scoring model.

The prediction model showed good discriminating power, as measured by the c-statistic of 0.852 in the original dataset and a mean c-statistic of 0.854 from the bootstrap samples (Figure 2). From the 500 bootstrap samples, the estimated average optimism in the original data was 0.002 (95% CI 0.001–0.003). The estimated discrimination of the model, penalizing for over fitting, was 0.850 (95% CI 0.849–0.851).

**Figure 3:**
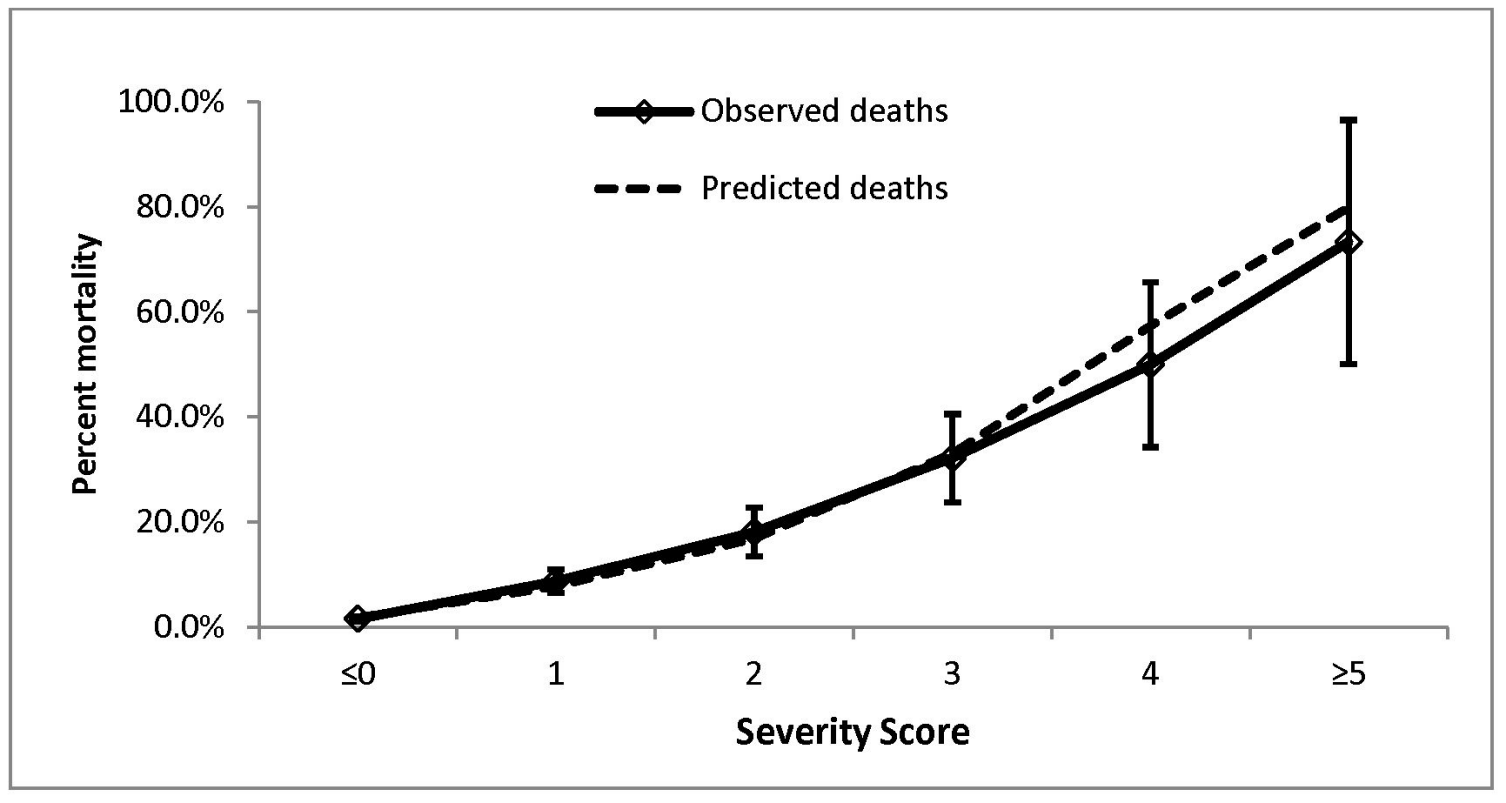
Observed mortality (with 95% CI) and mean predicted mortality by severity score.

The model also demonstrated good calibration (goodness-of-fit test p = 0.4705). Figure 3 includes a plot of percent observed mortality (with 95% CI) which very closely matches the mean predicted mortality by mRISC score. A graphical presentation of the predicted mortality for the period preceding and after February 2011 also shows a close match in the predictive power for the data collected in the two different time periods (Figure S1). The predicted mortality from the mRISC score in children <2 years of age was also similar to that for children aged ≥2 years (Figure S2).

## Discussion

We found that the mRISC scoring system accurately predicted the likelihood of fatal outcomes of children hospitalized for respiratory illness in Western Kenya using measurements taken at admission. In many Kenyan hospitals, where tools like radiography and laboratory testing are not commonly available, the mRISC score may prove a practical and objective tool for health care workers (Table 4) by helping to better guide decisions surrounding level of admission ward (i.e., high dependency units or intensive care unit vs. regular or low risk wards) and the prioritization of the use of limited resources such as high flow oxygen. The mRISC could also serve as a complementary tool to the IMCI guidelines, which are intended to help clinicians determine whether or not children need to be hospitalized, but which have less discriminating power for use in triage of hospitalized patients. Indeed, this is supported by the finding that while 17% of children classified as having severe/very severe pneumonia according to IMCI guidelines died in the hospital, 40% and 80% of children admitted with mRISC scores of ≥3 and ≥6, respectively, died in the hospital.

**Table 4:** Modified Respiratory Index of Severity in Children (mRISC) scoring.

Variable	Points
**History of present illness**	
Was the child **unconscious**? (Yes = 1 point; No = 0 points)	
Was the child **unable to drink/breastfeed**? (Yes = 1 point; No = 0 points)	
Did the child have **night sweats**? (Yes = −1 point; No = 0 points)	
**On physical examination**	
Does the child have **chest-wall in-drawing**? (Yes = 1 point; No = 0 points)	
Is the child **alert and awake**? (Yes = 0 point; No = 2 points)	
**Co-morbidities**	
Does the child have **malaria**? (Yes = −1 points; No = 0 points)	
Does the child have **malaria** and **chest-wall in-drawing**? (Yes = 1 point; No = 0 points)	
Is the child **dehydrated**? (Yes = 1 point; No = 0 points)	
Weight in Kg_________: Age in months__________	
z-score is **normal** or **high**(z-score>−2) = 0 points	
z-score is **low** or **very low** (z-score≤−2) = 1 point	
**Total points**	

Sub-optimal implementation of IMCI standards, coupled with fairly routine admission of children with uncomplicated malaria or recent *Mycobacterium tuberculosis* infection (TB), can together serve to lower the threshold for hospital admission and complicate patient triage and management within the hospital setting. This increases the chances of misclassifying subsets of children at low risk of severe outcomes. Such hospitalizations would take up resources that should have been focused on severe cases and therefore further underscores the need for a complementary tool such as the mRISC to help guide in-hospital triage. A recent study conducted in Kenya showed that clinicians were not universally following IMCI guidelines; 80% of the health workers had not been trained; and community members faced financial barriers in accessing services [8]. In Pakistan, even in situations where admissions with severe pneumonia as defined by IMCI were made, some of these children could have successfully been managed from home [23].

Our finding of the negative association between laboratory-confirmed malaria and risk of mortality among children hospitalized with respiratory illness suggests that these children may have had a lower threshold for admission and less severe disease and therefore less likely to die. This negative association is consistent with findings from a recent study conducted within the same area [24]. While this may highlight the value of rapid diagnosis of malaria, the same finding may not hold true in an area where malaria diagnosis is poorly sensitive or specific [25]–[27].

Although other studies have demonstrated increased mortality among HIV-infected patients with respiratory illness [28]–[30], we did not find this in our study. This could in part be explained by the fact that most of the patients who were HIV positive had either been started on cotrimoxazole or were on ARVs. Alternatively, this may be because we detected relatively few HIV-infected children, and this may have reduced our power to detect a difference in mortality. Also in contrast to other studies, low-oxygen saturation was not associated with increased mortality in the multivariable model. While unexpected, this makes the mRISC score more feasible to use in most health facilities in Kenya, where working pulse oximeters are not available. The observations that chest wall in-drawing, inability to drink or breastfeed and admission diagnosis of dehydration were only associated with mortality among children aged <2 years could in part be explained by the fact that these measures are most commonly made during the evaluation of very young children.

The study had several limitations. First, this study utilized data collected from only one site and as such further evaluation and external validation at other sites in Kenya should be undertaken. Secondly, it also possible that some of the children who were discharged alive may have had other serious complications or even died shortly afterwards, and are therefore misclassified in this analysis. Third, we did not include radiographic measurements in our analysis; it is conceivable that chest radiographic findings would have added discrimination if included in the scoring system, although this was not the case in South Africa [10]. Finally, as much as we adjusted for the type of drug treatment received and participation in clinical studies, other differences in care received could have be an unmeasured source of confounding.

In conclusion, this study shows that this adaptation of the RISC score initially developed in South Africa, may also have practical utility to rapidly identify children most at risk for fatal outcomes due to respiratory disease in rural Western Kenya. As a complementary tool for use alongside the IMCI guidelines, the mRISC could be useful in the triage of children with respiratory illness in the admission process and help improve their clinical management, and perhaps also serve as a standard measure of severity for use in epidemiological studies.

## Supporting Information

Figure S1 **Mean predicted percent mortality before and after February 2011 by severity score.**
(TIF)

Figure S2 **Mean predicted percent mortality before by age category.**
(TIF)
