# Supplementary figures and images for: Predicting Mortality among Hospitalized Children with Respiratory Illness in Western Kenya, 2009–2012
Source: PLoS One. 2014 Mar 25;9(3):e92968. doi: 10.1371/journal.pone.0092968 (PMC3965502; doi:10.1371/journal.pone.0092968)

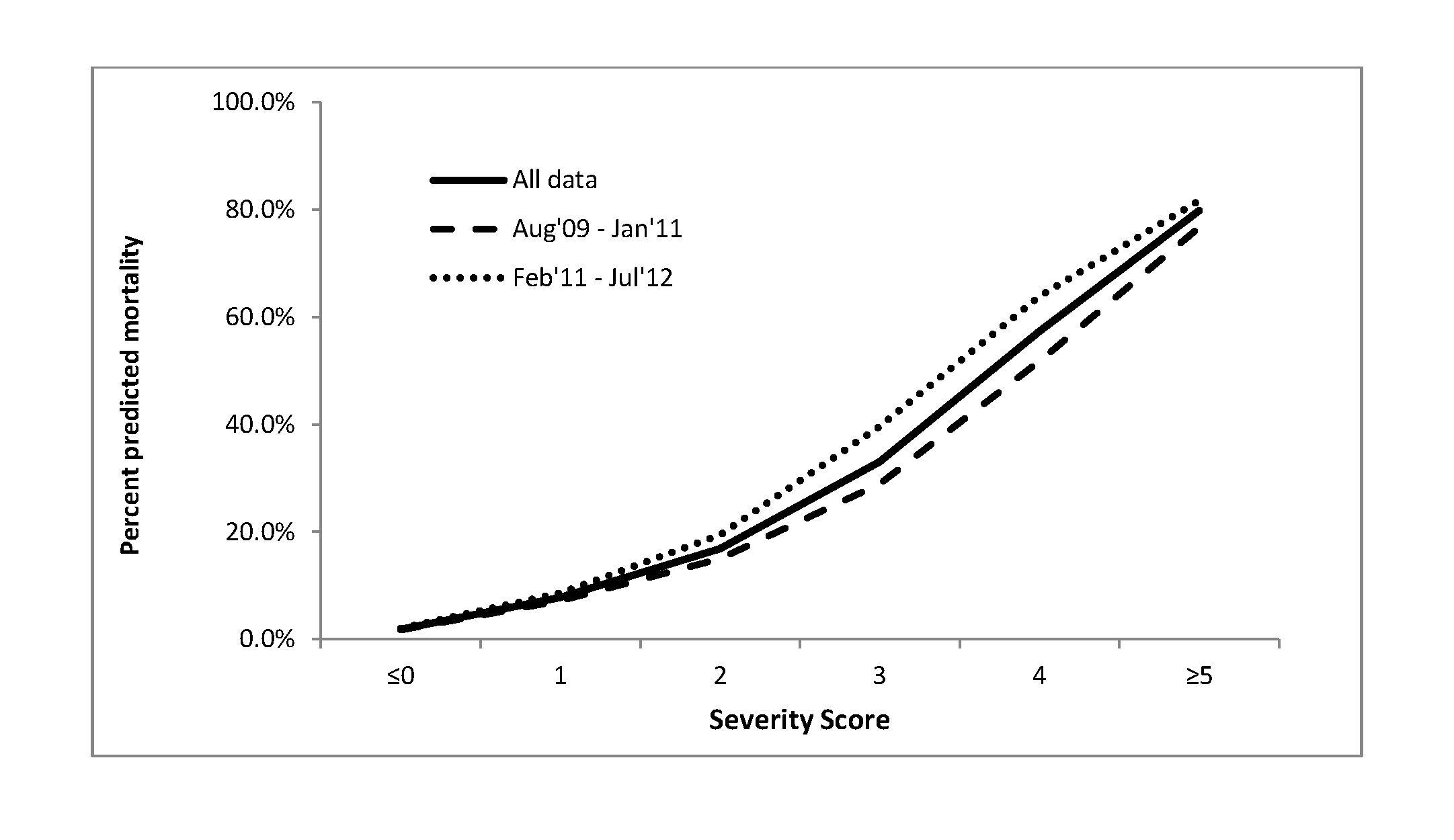

Supplement: Figure S1 — Mean predicted percent mortality before and after February 2011 by severity score. (TIF) [file pone.0092968.s001.tif]

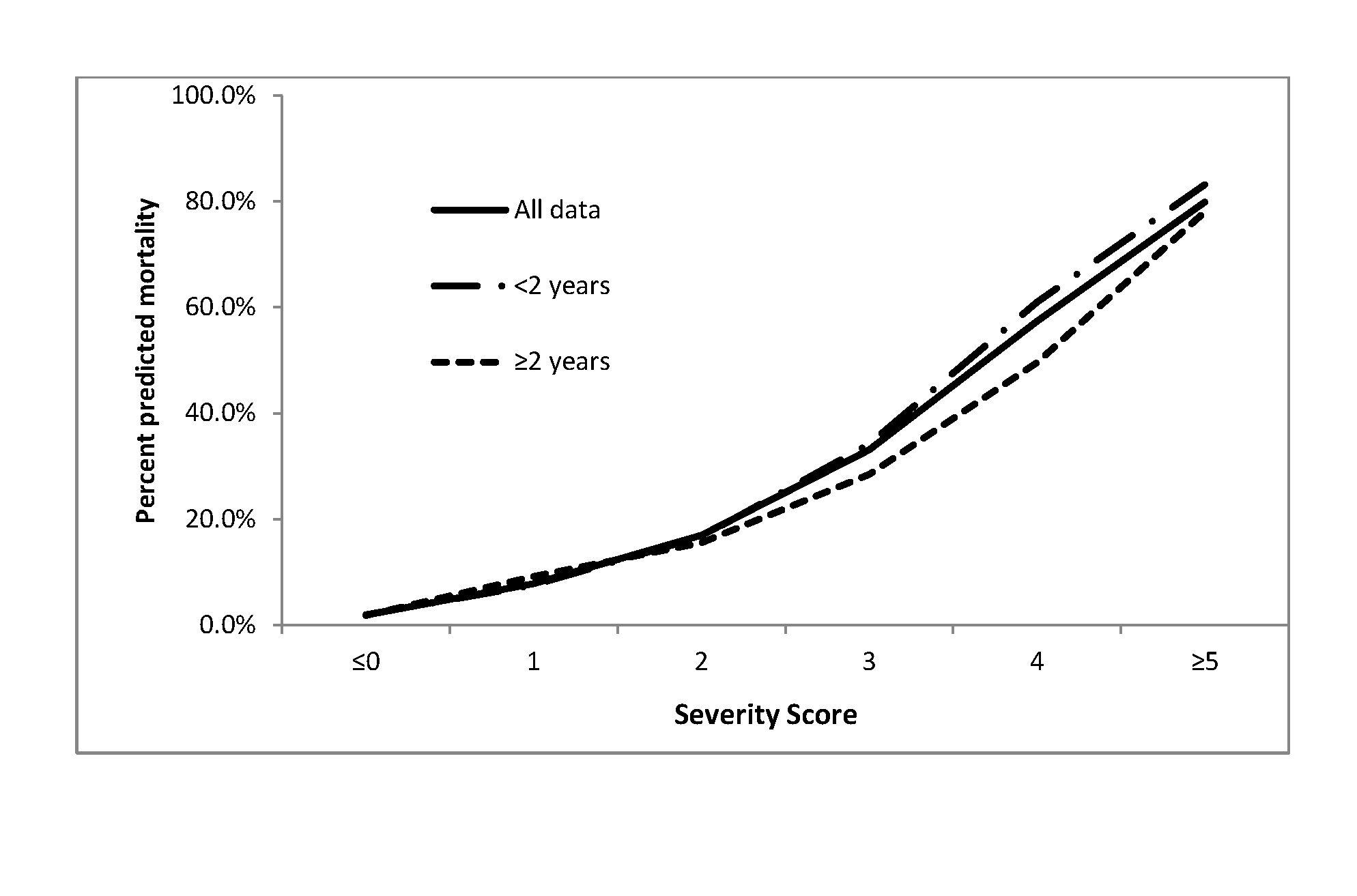

Supplement: Figure S2 — Mean predicted percent mortality before by age category. (TIF) [file pone.0092968.s002.tif]
